# Mild Endoplasmic Reticulum Stress Protects Against Lipopolysaccharide-Induced Astrocytic Activation and Blood-Brain Barrier Hyperpermeability

**DOI:** 10.3389/fncel.2018.00222

**Published:** 2018-07-27

**Authors:** Yiwei Wang, Yinan Chen, Qin Zhou, Jiawen Xu, Qingqing Qian, Pengfei Ni, Yanning Qian

**Affiliations:** Department of Anesthesiology, The First Affiliated Hospital of Nanjing Medical University, Nanjing, China

**Keywords:** endoplasmic reticulum stress, astrocytes, hormesis, blood-brain barrier, neuroinflammation, neurodegeneration

## Abstract

Recent research has revealed that uncontrolled chronic neuroinflammation is closely associated with diverse neurodegenerative diseases, by impairing blood-brain barrier (BBB) function and astrocytic reaction. Endoplasmic reticulum (ER) stress is conventionally linked to the loss of neuronal structure and function and should be widely attenuated. This notion has been questioned by recent experimental studies, which have shown that non-harmful levels of ER stress had numerous beneficial roles against neurodegeneration, including neuroprotection and inhibition of cytokine production. Here, we investigated the mild ER stress-based regulation of LPS-induced inflammatory responses in astrocytes. Primary astrocytes were exposed to tunicamycin (TM), a compound that activates ER stress, with or without the ER-stress inhibitor sodium 4-phenylbutyrate (4-PBA) before LPS treatment. Astrocytic activation, proinflammatory factor production, and the extent of ER stress were assessed. In addition, the effect of mild ER stress on astrocytes and BBB function was determined *in vivo*. Male Sprague-Dawley rats received intracerebroventricular injections of TM with or without intraperitoneal 4-PBA before LPS administration. The levels of astrocytic activation and BBB permeability were measured after treatment. Our results showed that lower doses of TM resulted in a mild ER-stress response without inducing cytotoxicity and tissue toxicity. Non-toxic ER-stress preconditioning ameliorated LPS-induced overactivation and inflammatory responses in astrocytes. Moreover, pre-exposure to non-lethal doses of TM improved LPS-induced BBB impairment and cognitive ability dysfunction in rats. However, 4-PBA, reversed the protective effect of TM preconditioning *in vitro* and *in vivo*. We conclude that mild ER stress (“preconditioning”) can alleviate LPS-induced astrocytic activation and BBB disruption. Our findings provide a better understanding for the regulatory role of ER stress in neuroinflammation and indicate that mild ER stress might have therapeutic value for the treatment of neurodegenerative diseases.

## Introduction

Neurodegeneration is characterized by neuronal dysfunction and death in the central nervous system (CNS) (Estes and McAllister, 2016). Emerging evidence indicates that sustained inflammatory responses in the CNS associated with blood-brain barrier (BBB)-disruption contribute to neurodegenerative-disease progression (Glass et al., 2010; Broux et al., 2015). Systemic injection of lipopolysaccharide (LPS), a complex molecule containing both lipid and polysaccharide parts, can elicit neuroinflammation in the normal rat brain (Pintado et al., 2012). Animals with LPS-induced neuroinflammation are therefore frequently used as an experimental model for neurodegeneration and cognitive disorders (Qin et al., 2007).

Considering the many essential functions of astrocytes in the healthy CNS, astrocytic dysfunction might participate in the propagation and regulation of neuroinflammation. Astrocytes become activated in response to various stimuli, from subtle inflammatory changes in their microenvironment to massive neuronal damage (Sofroniew, 2015). Reactive astrocytes appear to play vital roles in mediating the production of proinflammatory effector molecules and amplifiers, such as chemokines, cytokines, and reactive oxygen species. These influence the state of surrounding cells (e.g., neurons, microglia, and other astrocytes), thereby triggering neuroinflammation leading to exacerbation of neurotoxicity and neurodegeneration (Gorina et al., 2011).

At least one-third of all proteins in the cell are first synthesized, folded, and structurally mature in the endoplasmic reticulum (ER); then, they are transported to the membrane compartment (Hetz and Mollereau, 2014). In astrocytes, the ER acquires a central role in sensing cellular stress after protein unfolding/misfolding in its lumen (known as ER stress), with the consequent activation of a cellular response termed the “unfolded protein response” (UPR). UPR is initiated by three principal stress sensors: the inositol-requiring protein 1α (IRE1α)-spliced X-box-binding protein 1 (XBP1s) pathway, the activating transcription factor (ATF)-6α pathway, and the protein kinase RNA-like ER kinase (PERK)-eukaryotic translation initiation factor 2α (EIF2α) pathway (Martin-Jiménez et al., 2017).

Endoplasmic reticulum stress in astrocytes has sometimes been viewed as a response mainly contributing to neurodegeneration, which should therefore be broadly attenuated (Martin-Jiménez et al., 2017). This notion is refuted by rapidly growing evidence, which shows that non-harmful levels of ER stress exert numerous beneficial functions that improve outcomes in CNS inflammation (Hetz and Mollereau, 2014; Matus et al., 2014). These findings have been reinforced by an array of related studies. A recent study showed that pretreatment with ER stressors protected against amyloid-β toxicity to reverse memory impairment in Alzheimer’s disease (AD) (Casas-Tinto et al., 2011). Similarly, maintaining ER stress at a moderate level inhibits neuronal death in mouse and *Drosophila* Parkinson disease (PD) models (Fouillet et al., 2014). It is significant to emphasize that the roles of ER stress in neurodegenerative conditions are not stereotypically linear, but instead context-specific and complex, as determined by the type, severity, time, and duration of the insult. Specific mechanisms need to be identified and addressed.

In cardiac surgery, myocardial ischemic preconditioning is favorable for improving heart function. There is growing awareness that low perturbations of ER function also trigger a hormetic response, known as “ER hormesis,” which involves a biologically-favorable response induced by exposing a cell/organism to non-lethal pharmacological ER stressors (Matus et al., 2014). However, there have so far been few scientific studies on the involvement of ER hormesis in astrocytes. Therefore, in the current study, we aimed to elucidate the effects of mild ER stress on LPS-induced inflammatory responses in astrocytes and to investigate whether mild ER stress exerts a protective effect against LPS-induced BBB hyperpermeability.

## Materials and Methods

### Animals

One hundred and seventy-four male Sprague-Dawley (SD) rats (weight: 180–220 g) were used in the current study. All experiments were performed according to the ethical guidelines of the National Institutes of Health (NIH) guide for the Care and Use of Laboratory animals (NIH Publications No. 8023, revised 1978) and approved by the Nanjing Medical University Animal Care and Use Committee (IACUC-14030126). The rats were housed under standardized conditions (12-h light/dark cycle, 22.0 ± 1.0°C, and 40% humidity), and water and food were provided *ad libitum*. Based on the 3R principle for the ethical use of animals in research, all efforts were made to reduce animal use and suffering.

### Intracerebroventricular Cannula Implantation

Rats were implanted with an indwelling lateral intracerebroventricular (icv) catheter for brain injection of drugs as previously described (Zhang et al., 2016). The animals were anesthetized (isoflurane, 2.1% inspired concentration in 0.3 FiO2) and placed on a stereotaxic apparatus. Stereotaxic coordinates for the placement of the icv cannulas were 0.8 mm rostral, 1.5 mm lateral to the bregma, and 3.7 mm ventral from the dorsal surface of the skull. Rats were individually housed after surgery and handled daily to familiarize the animals with the investigators and to check the guide catheter. Rats were allowed a minimum of 14 days to recover prior to icv administration of pharmacological agents and/or other experimental measurements.

### Behavioral Tests

Trace fear conditioning, contextual assessment, and the Y-maze test were performed. The molecular-biology tests and the behavioral experiments were conducted using different animals. The behavioral test design is briefly illustrated in Supplementary Figure 1A. The specific methods can be found in the Supplementary Material.

### Drug Administration

The well-known ER-stress inducer tunicamycin (TM) was diluted in sterile saline containing 10% dimethyl sulfoxide (DMSO) to create a stock concentration of 15 μg/μl. Then, 4-phenylbutyric acid (4-PBA), a common ER-stress inhibitor, was diluted with sterile saline to a concentration of 10 mg/ml and injected intraperitoneally (ip) at a dose of 100 mg/kg according to a previous study (Wang et al., 2017). LPS was dissolved in sterile saline to the concentration of 50 μg/ml. Each rat was ip-injected at a dose of 500 μg/kg, according to our previous study (Sun et al., 2015).

### Experimental Protocol and Pharmacological Treatments

#### Experiment 1 (*In Vivo*)

Rats were divided into five groups (groups A–E), with 16 animals in each group. Empty treatments were administered to the rats in group A. Rats received either icv injections of 2 μl of vehicle (group B), 2 μl of 0.15 μg/μl TM (group C), 2 μl of 1.5 μg/μl TM (group D), or 2 μl of 15 μg/μl TM (group E). These concentrations were based on previous work from our laboratory (Wang et al., 2017). The study design is briefly illustrated in Supplementary Figures 1B,C.

#### Experiment 2 (*In Vivo*)

Rats were randomly divided into five groups (groups A–E), with 16 animals in each group. Rats in groups B, C, D, and E received icv cannula implantation, while empty treatments were administered to the rats in group A. Rats were administered an intraperitoneal injection of 100 mg/kg 4-PBA (group E) or an equivalent volume of sterile saline (groups B–D). Next, a dose of 2 μl of 1.5 μg/μl TM (groups D and E) or 2 μl of vehicle (10% DMSO in saline) (group B and C) was injected through the brain cannulas. One hour after 4-PBA/TM treatment, intraperitoneal injections were administered (group B: normal saline; groups C, D, and E: 500 μg/kg LPS). The experimental design is briefly illustrated in Supplementary Figures 1D,E.

According to the “3R” principle of ethical animal use, the same rats were used for group A in both experiments 1 and 2.

#### Experiment 1 (*In Vitro*)

Primary astrocytes were incubated for 24 h at different concentrations of TM (0.1, 1, and 10 ng/ml). The study design is briefly shown in Supplementary Figure 1F.

#### Experiment 2 (*In Vitro*)

Astrocytes were pretreated transiently with TM at 1 ng/ml with or without 4-PBA for 1 h, followed by thorough washing to remove the TM and 4-PBA, and incubated in fresh medium with or without LPS for 24 h. The experimental procedure is briefly illustrated in Supplementary Figure 1G.

### Primary Astrocyte Cultures

Primary astrocyte cultures were prepared from neonatal (P1–P2) rat brains as previously described (Xu et al., 2018). Briefly, the cerebral cortices were isolated and digested at 37°C for 10 min in phosphate-buffered saline (PBS) containing 0.25% trypsin-EDTA. The dissociated cells were passed through a 100-μm pore mesh to remove debris. The cell suspension was seeded on poly-D-lysine-coated flasks in high-glucose Dulbecco’s Modified Eagle’s medium supplemented with 10% fetal bovine serum. The culture medium was changed for fresh solution every 3 days. After 10 days of cultivation, astrocytes were separated from the microglia by shaking for 5 h at 150 rpm. The purity of the astrocytes was >95% as confirmed by anti-GFAP immunochemical staining.

### Cell Counting Kit-8 (CCK-8) Assay

Astrocytic viability was quantified using a CCK-8 assay based on the manufacturer’s instructions (Beyotime, Shanghai, China). Briefly, 10 μl of the CCK-8 solution was added to each well of a 96-well plate containing 3 × 10^4^ astrocytes following treatment with different concentrations of reagents and the absorbance was measured after a 1-h incubation at 37°C at 450 nm using a DTX-880 multimode microplate reader. Each experiment was repeated at least three times.

### Enzyme-Linked Immunosorbent Assay (ELISA)

IL-6 and IL-1β levels in the astrocytic supernatant were measured using ELISA kits from R&D Systems (Minneapolis, MN, United States) according to the manufacturer’s instructions. See the Supplementary Material for more information.

### Western Blot Analysis

Hippocampal tissues and astrocytes were homogenized in RIPA lysis buffer, which contained 50 mM Tris, 150 mM NaCl, 1% Triton X-100, 2 mM EDTA, 1.5 μg/mL leupeptin, and 1 mM phenylmethylsulfonyl fluoride. The lysate was centrifuged for 20 min at 12,000 × *g* at 4°C. The protein content was determined by BCA assay (Thermo Scientific, Waltham, MA, United States), and 20 μg of protein was loaded per lane on a modified sodium dodecyl sulfate polyacrylamide gel electrophoresis. Separated proteins were transferred onto polyvinylidene difluoride membranes (Milipore, Bedford, MA, United States) and blocked for 1 h with 5% non-fat milk in Tris-buffered saline with Tween 20. Blocked membranes were probed overnight with specific primary antibodies diluted in 5% non-fat milk according to the recommendation of the manufacturer. After washing, membranes were incubated with anti-rabbit or anti-mouse IgG-HRP secondary antibodies, washed, and incubated with ECL reagent before exposure to film. Densitometry analysis was performed with the Image Lab software (Bio-Rad, Richmond, CA, United States) and quantified using the gel analysis plugin for Image J (NIH, Bethesda, MD, United States).

### Immunohisto/Cytochemistry

The astrocyte culture medium was removed, and cells were fixed with 4% paraformaldehyde (PFA) for 30 min. The cells were then blocked with 5% bovine serum albumin containing 0.1% Triton X-100 for 1 h. Specific primary antibodies were added at an indicated concentration and incubated overnight at 4°C. Following washes with PBS, the secondary antibodies (Alexa-Fluor-conjugated) were added and incubated for 1 h at 37°C. The images were captured using a confocal microscope (Zeiss LSM 510; Zeiss, Oberkochen, Germany).

Immunohistochemistry was used to determine the activation of the astrocytes in the hippocampus. Briefly, rats were perfused transcardially with 37°C saline followed by ice-cold 4% PFA. The ipsilateral hippocampal tissue was removed and fixed for 1 h in 4% PFA at 4°C. The tissue was sectioned at 15 μm with a cryostat. Sections were incubated overnight with specific primary antibodies followed by the appropriate secondary antibodies. Confocal images were obtained with a confocal microscope (Zeiss LSM 510).

The confocal microscope parameters were maintained constant across all samples, and samples of different groups were always processed in parallel. Fluorescence intensity was quantified using ImageJ and normalized to the fluorescence levels observed in untreated samples as described.

### Evan’s Blue (EB) Extravasation

Two percent of EB dye (Sigma-Aldrich, St. Louis, MI, United States) in 0.9% saline (5 mL/kg) was injected intravenously (right femoral vein), and 30 min later, the rats were perfused transcardially with 0.9% saline to remove the intravascular blood and EB. Then, the brains were harvested and homogenized in 0.5 ml of 50% trichloroacetic acid and centrifuged at 10,000 × *g* for 10 min. EB concentrations in supernatants were measured using spectrophotometry at 620 nm.

### Statistical Analysis

Data are expressed as mean ± SEM. Two-tailed unpaired *t*-tests were used for comparisons between two groups, and multiple comparisons were evaluated using an appropriate ANOVA. *Post hoc* comparisons were performed using Tukey’s test when appropriate. The alpha level was set at *P* < 0.05.

## Results

### Low Concentrations of TM Generated Non-toxic, Mild ER Stress in Astrocytes

**FIGURE 1:**
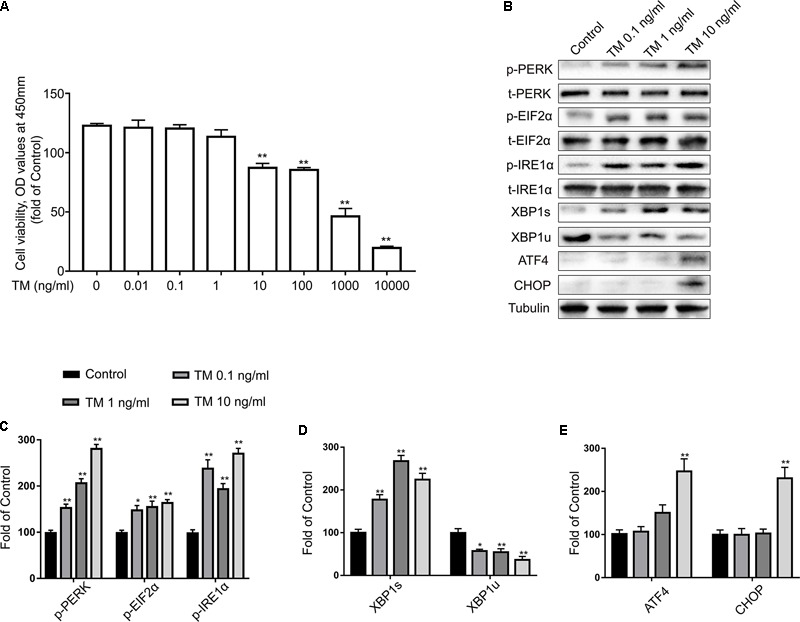
Low doses of TM activated a non-toxic, mild ER stress in primary cultured astrocytes. **(A)** Primary astrocytes were treated with TM (0.01 to 10000 ng/ml) for 24 h followed by assessment of cell viability using the CCK-8 assay. **(B)** The expression levels of p-PERK, p-EIF2α, p-IRE1α, XBP1s, XBP1u, ATF4, and CHOP in primary cultured astrocytes were detected by Western blotting using specific antibodies. **(C)** Phosphorylated levels of PERK, EIF2α, and IRE1α were quantified and normalized to corresponding total levels. **(D)** Expression of XBP1s and XBP1u was quantified and normalized to Tubulin expression. **(E)** Expression of ATF4 and CHOP was quantified and normalized to Tubulin expression. Each value was then expressed relative to that of the control group, which was set to 100. All experiments were repeated three times. ^∗^*P* < 0.05, ^∗∗^*P* < 0.01 vs. control group. The data are presented as the mean ± SEM.

Many studies have shown that astrocytes may be vulnerable to ER stress-induced cell apoptosis. Consequently, primary cultured astrocytes were first exposed to different concentrations of TM for 24 h and their activities were tested using the CCK8 reagent. Our results indicate that TM (<10 ng/ml) had no effects on the cell viability of astrocytes compared to the control group (**Figure 1A**). Therefore, doses of 0.1, 1, and 10 ng/ml were selected for the subsequent experiments. The next experiment was conducted to explore whether lower concentrations of TM could also cause mild ER stress in astrocytes.

IRE1α initiates the most conservative signaling pathway of ER stress that, upon phosphorylation, cuts the non-conventional splicing of XBP-1 (XBP1u) mRNA into spliced-XBP1 (XBP1s) mRNA, which encodes a transcription activator that regulates ER protein folding (Gardner et al., 2013). Thus, to further determine the extent of IRE1-signaling activation, we measured the protein levels of p-IRE1α, XBP1s, and XBP1u using western blot analysis with specific antibodies (**Figure 1B**). TM (0.1, 1, and 10 ng/ml) significantly increased the expression levels of p-IRE1α and XBP1s, while the same treatment reduced XBP1u expression (**Figures 1C,D**). As a crucial stress sensor of ER stress, PERK is a transmembrane kinase that phosphorylates EIF2α at Ser51(Gardner et al., 2013). However, persistent EIF2α phosphorylation paradoxically upregulates translation of ATF4 mRNA, which in turn activates pro-apoptotic components such as the transcription of CCAAT/enhancer binding protein (C/EBP) homologous protein (CHOP) (Hetz and Mollereau, 2014).

To characterize the activation kinetics of PERK signaling of ER stress, we examined the expression of p-PERK and its downstream products: p-EIF2α, ATF4, and CHOP using western blot analysis. Compared with the control group, p-PERK and p-EIF2α phosphorylation was modestly elevated at concentrations of 0.1, 1, and 10 ng/ml TM (**Figure 1C**). It is worth noting that lower doses of TM (0.1 and 1 ng/ml) were no longer effective at increasing ATF4 and CHOP expression levels but that high concentrations (10 ng/ml) did increase ATF4 and CHOP expression (**Figure 1E**).

**FIGURE 2:**
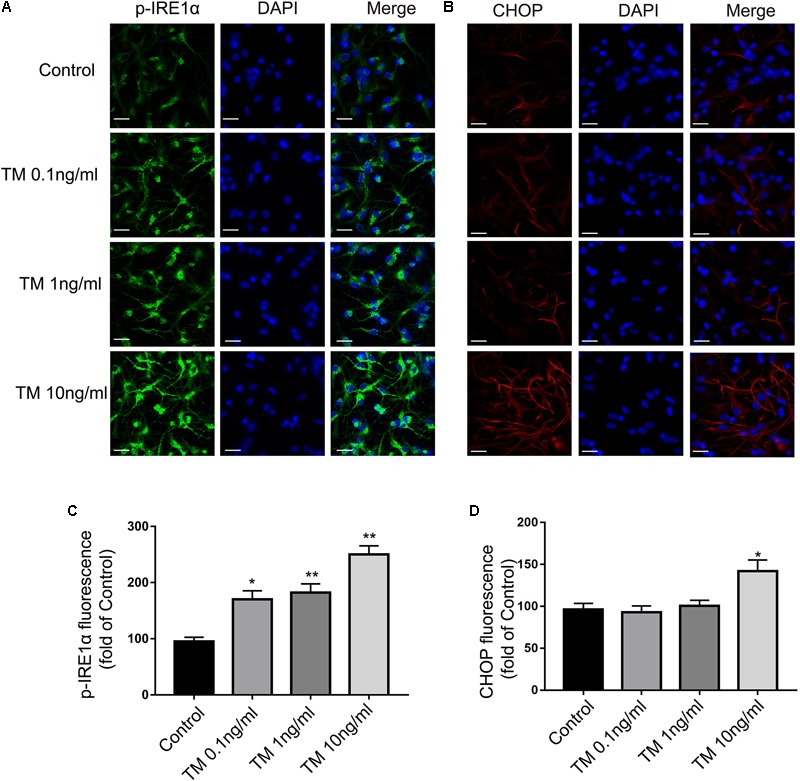
Mild ER stress increased p-IRE1α expression but had no effect on CHOP expression in primary cultured astrocytes. **(A)** Astrocytes were stained with p-IRE1α antibody. p-IRE1α-immunopositivity in primary astrocytes was observed using confocal scanning. **(B)** Astrocytes were stained with CHOP antibody. CHOP-immunopositivity in primary astrocytes was observed using confocal scanning. Blue staining represents DAPI. Scale bar = 25 μm. **(C)** Quantitative data of the mean intensity of p-IRE1α fluorescence in primary astrocytes. **(D)** Quantitative data of the mean intensity of CHOP fluorescence in primary astrocytes. Each value was then expressed relative to that of the control group, which was set to 100. All experiments were repeated three times. ^∗^*P* < 0.05, ^∗∗^*P* < 0.01 vs. control group. The data are presented as the mean ± SEM.

Similar results were also observed in the immunofluorescence assay (**Figures 2A,B**). Consistent with the previous data, increases of IRE1α phosphorylation were observed following TM treatment (0.1, 1, and 10 ng/ml) in the primary cultured astrocytes (**Figure 2C**). CHOP levels, in contrast, were not changed in primary cultured astrocytes treated with TM at doses of 0.1 or 1 ng/ml. The CHOP expression was only elevated in response to the highest dose of TM (10 ng/ml) (**Figure 2D**). These findings showed that lower concentrations of TM (0.1 and 1 ng/ml) triggered benign and mild ER-stress responses in the astrocytes.

### Mild ER Stress Attenuated LPS-Induced Astrocytic Inflammatory Responses and Overactivation

We observed that low concentrations of TM (0.1 and 1 ng/ml) caused mild ER stress in the astrocytes but did not induce cell death. In the following experiment, we therefore selected a dosage of TM (1 ng/ml) to generate mild ER stress in the astrocytes. Next, we investigated the effects of mild ER stress in LPS-stimulated astrocyte activation.

#### TM Inhibited IL-1β and IL-6 Production in Primary Cultured Astrocytes

**FIGURE 3:**
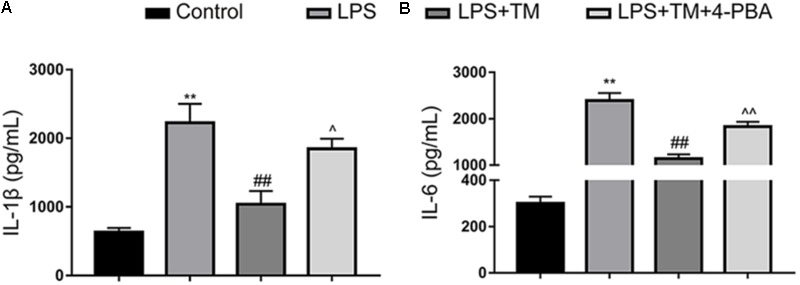
Mild ER stress inhibits proinflammatory cytokine IL-1β and IL-6 production in primary cultured astrocytes. **(A)** The levels of the proinflammatory factors IL-1β were detected by ELISA. **(B)** The levels of the proinflammatory factors IL-6 were detected by ELISA. The data are representative of 3 independent experiments. ^∗^*P* < 0.05, ^∗∗^*P* < 0.01 vs. control group. ^#^*P* < 0.05, ^##^*P* < 0.01 vs. LPS treatment group. ^∧^*P* < 0.05, ^∧∧^*P* < 0.01 vs. TM treatment group. The data are presented as the mean ± SEM.

Astrocytes were treated with LPS in the absence or presence of TM. Because astrocytes participate in neuroinflammation via the excessive secretion of proinflammatory factors, IL-6 and IL-1β were analyzed with ELISA. As the results shown in **Figure 3** illustrate, LPS enhanced the expression of IL-1β and IL-6. Additionally, 1 ng/ml TM did not enhance, but instead suppressed, LPS-induced proinflammatory factor expression.

#### TM Reversed LPS-Induced Astrocyte Activation

**FIGURE 4:**
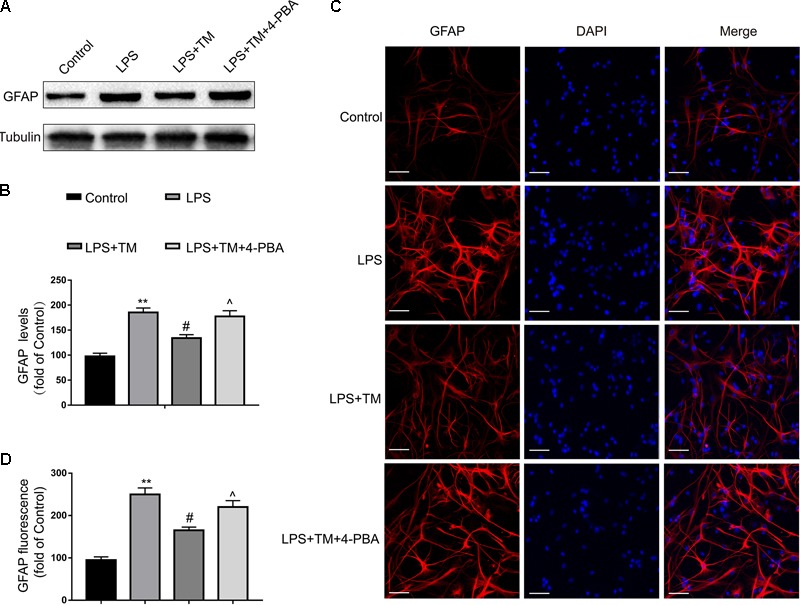
Mild ER stress alleviated astrocyte activation. **(A)** The protein levels of GFAP were detected by Western blotting using specific antibody in the primary astrocytes. **(B)** Expression of GFAP was quantified and normalized to Tubulin levels. Each value is expressed relative to that in the control group, which was set to 100. **(C)** Astrocyte was stained with GFAP antibody as indicated. Blue staining represents DAPI. Scale bar = 25 μm. **(D)** Quantitative data of the mean intensity of GFAP fluorescence in primary astrocytes. Each value is expressed relative to that in the control group, which was set to 100. All experiments were repeated three times. ^∗^*P* < 0.05, ^∗∗^*P* < 0.01 vs. control group. ^#^*P* < 0.05, ^##^*P* < 0.01 vs. LPS treatment group. ^∧^*P* < 0.05, ^∧∧^*P* < 0.01 vs. TM treatment group. The data are presented as the mean ± SEM.

To further confirm the protective effects of TM on primary astrocytes, GFAP expression levels corresponding to activated astrocytes were tested with western blot analysis. LPS significantly increased the expression of GFAP in primary cultured astrocytes compared with the levels observed in the control group, but these elevations were remarkably inhibited by TM pretreatment (**Figures 4A,B**). To validate these findings, astrocytes were labeled with GFAP by immunofluorescence (**Figures 4C,D**), which also confirmed that TM can inhibit LPS-induced astrocytic activation.

#### 4-PBA Reversed TM-Induced Suppression of Astrocytic IL-1β, IL-6, and GFAP Production

Next, we examined whether mild ER-stress activation is responsible for TM-mediated inhibition of astrocytic activation and anti-inflammatory responses.

**FIGURE 5:**
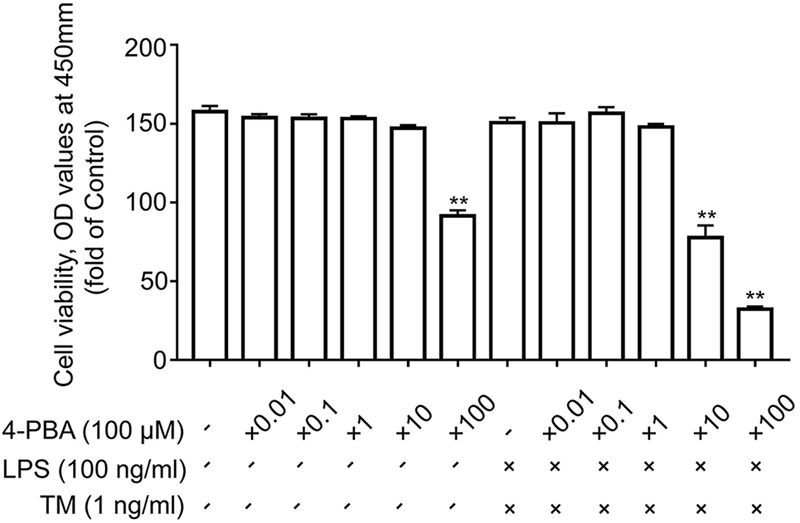
The effects of 4-PBA on cell viability in primary astrocytes. Primary cultured astrocytes were subjected to LPS and TM and treated with the indicated dosage of 4-PBA. After 24 h of treatment, cell viability was determined using CCK-8. All experiments were repeated three times. ^∗^*P* < 0.05, ^∗∗^*P* < 0.01 vs. control group. The data are presented as the mean ± SEM.

**FIGURE 6:**
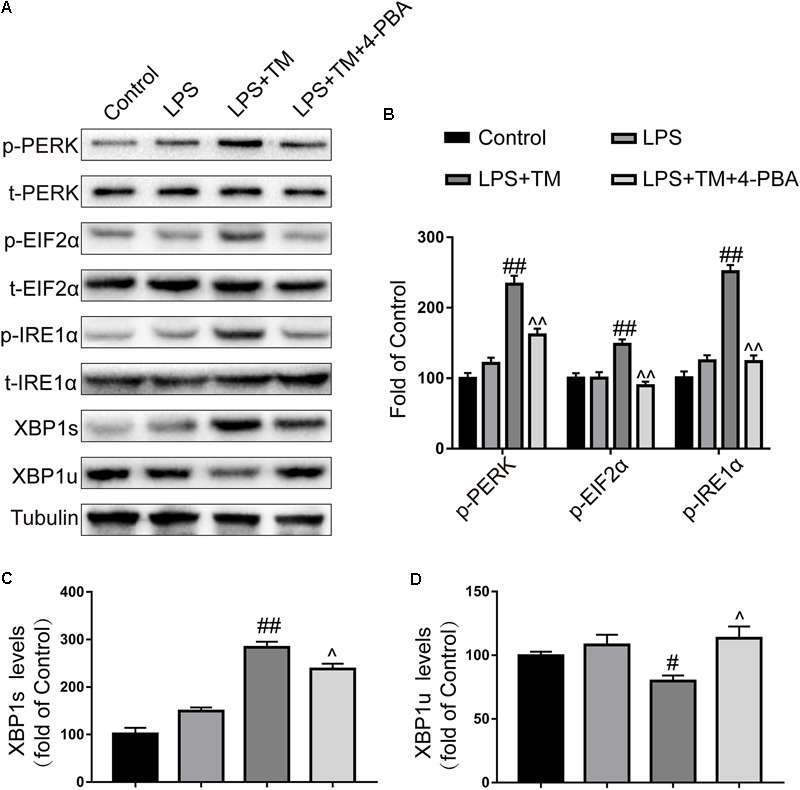
4-PBA partially inhibited mild ER stress in primary cultured astrocytes. **(A)** The expression levels of p-PERK, p-EIF2α, p-IRE1α, XBP1s, and XBP1u in primary cultured astrocytes were detected by Western blotting using specific antibodies. **(B)** Phosphorylated levels of PERK, EIF2α, and IRE1α were quantified and normalized to corresponding total levels. **(C)** Expression of XBP1s was quantified and normalized to Tubulin expression. **(D)** Expression of XBP1u was quantified and normalized to Tubulin expression. Each value is expressed relative to that in the control group, which was set to 100. All experiments were repeated three times. ^#^*P* < 0.05, ^##^*P* < 0.01 vs. LPS treatment group. ^∧^*P* < 0.05, ^∧∧^*P* < 0.01 vs. TM treatment group. The data are presented as the mean ± SEM.

To test this hypothesis, we used a chemical chaperone, 4-PBA, to reduce ER stress. Astrocytes were first subjected to TM (1 ng/ml), individually or in the presence of 4-PBA at progressive concentrations for 1 h before treatment with LPS (100 ng/ml) for 24 h. Our results indicate that treatment of the primary cultured astrocytes with 1 ng/ml TM and 100 ng/ml LPS in the presence of T 4-PBA (<1,000 μM) had no significant cytotoxicity (**Figure 5**). We therefore selected a dose of 100 μM 4-PBA to ameliorate ER stress in the astrocytes. The primary cultured astrocytes were subjected to TM (1 ng/ml) and 4-PBA (100 μM) treatment for 1 h. Reperfusion was then performed by refreshing the astrocytes with normal medium, containing 100 ng/ml LPS. As shown in **Figure 6A**, there was a clear effect of 4-PBA against ER stress, as it obviously decreased p-PERK, p-EIF2α, p-IRE1α, and XBP1s protein levels and increased XBP1u protein levels compared to non-PBA treated cells (**Figures 6B–D**).

Importantly, 4-PBA partially blocked the TM-mediated inhibition of astrocytic activation and anti-inflammatory effects, as reflected by the increased expression of IL-1β, IL-6, and GFAP. We wondered whether this opposite effect was caused by the cytotoxic effects of 4-PBA.

Using the CCK-8 method, we excluded this possibility, as coincubation with LPS (100 ng/ml), TM (1 ng/ml), and 4-PBA (100 μM) for 24 h revealed no significant cytotoxicity and did not affect cell viability (**Figure 5**). Collectively, these data demonstrate that TM pretreatment protected astrocytes by, at least partly, inducing mild ER stress.

### Mild ER Stress Ameliorated LPS-Induced Cognitive Decline

To confirm the *in vitro* findings, we also examined the effects of mild ER stress *in vivo*.

Our previous study showed that a 3-μg dose of TM triggered mild and benign perturbations of ER function in the rat hippocampus but did not induce drastic behavioral alterations or death (Wang et al., 2017). In the following experiment, rats were administered an icv injection of 3 μg TM to induce mild ER stress followed by systemic LPS administration.

**FIGURE 7:**
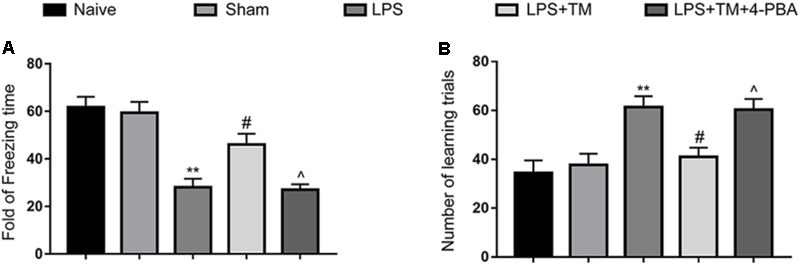
Mild ER stress ameliorated LPS-induced cognitive impairment in the hippocampus. **(A)** Contextual fear response, as measured by freezing time, was determined in the rats (*n* = 12). **(B)** The number of learning trials was recorded to analyze the Y-maze test (*n* = 12). The data are presented as the mean ± SEM. ^∗^*P* < 0.05, ^∗∗^*P* < 0.01 vs. naïve group. ^#^*P* < 0.05, ^##^*P* < 0.01 vs. LPS treatment group. ^∧^*P* < 0.05, ^∧∧^*P* < 0.01 vs. TM treatment group. The data are representative of three independent experiments.

Consistent with our previous experiment, LPS caused severe memory impairment in rats, as reflected by a significant reduction in freezing behavior and an improvement in the number of learning trials (Sun et al., 2015; Wang et al., 2017). Notably, TM pretreatment ameliorated LPS-induced cognitive decline. However, concomitant administration of 4-PBA partially reversed the cognitive recovery conferred by TM (**Figure 7**). Therefore, these results further substantiate our previous findings that mild ER stress can protect against LPS-induced cognitive dysfunction.

### Mild ER Stress Attenuated LPS-Induced Astrocytic Activation in the Hippocampus

**FIGURE 8:**
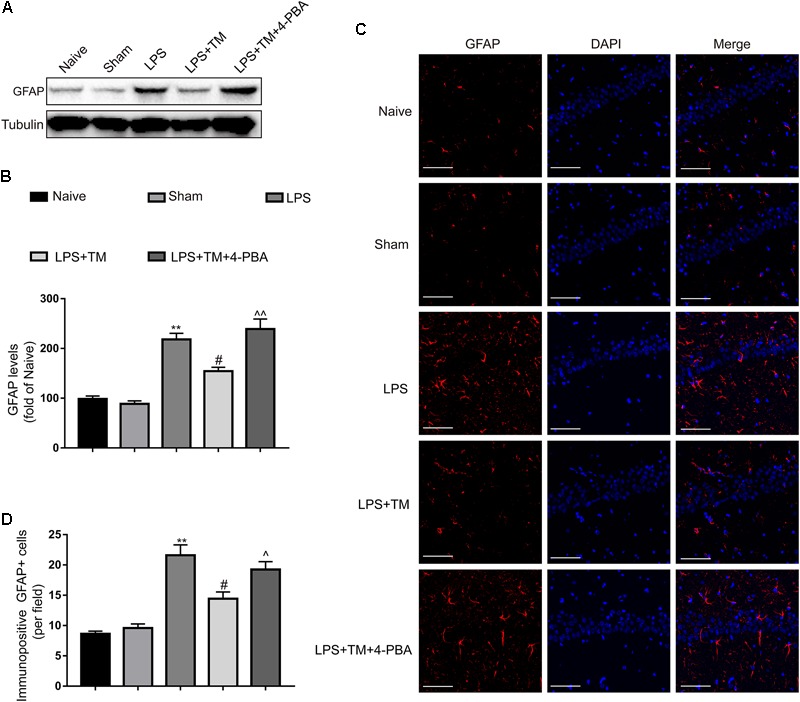
Mild ER stress reduced the hippocampal astrocyte activation. **(A)** The protein levels of GFAP were detected by Western blotting using specific antibody in hippocampus. **(B)** Expression of GFAP was quantified and normalized to Tubulin levels. Each value was expressed relative to that of the naïve group, which was set to 100 (*n* = 6). **(C)** Immunofluorescent staining was used to detect GFAP, a maker of astrocytes, in hippocampal CA1 region. Blue staining represents DAPI. Scale bar = 200 μm. **(D)** Quantitative of GFAP-positive cells in the CA1 area of hippocampus. The data are representative of three independent experiments. ^∗^*P* < 0.05, ^∗∗^*P* < 0.01 vs. naïve group. ^#^*P* < 0.05, ^##^*P* < 0.01 vs. LPS treatment group. ^∧^*P* < 0.05, ^∧∧^*P* < 0.01 vs. TM treatment group. The data are presented as the mean ± SEM.

The western blot analysis showed that systemic LPS administration enhanced the expression of GFAP in the hippocampi of rats, but these elevations were potently suppressed by TM pretreatment. Similar to the *in vitro* results, 4-PBA diminished the TM-mediated inhibition of astrocytic activation in the hippocampus (**Figures 8A,B**). These western blot findings were further validated by immunofluorescence (**Figures 8C,D**). Our results therefore further confirmed that mild ER stress was required for the TM-mediated protection.

### Mild ER Stress Alleviated LPS-Induced BBB Hyperpermeability

#### Mild ER Stress Counteracted LPS-Induced Albumin Leakage in the Hippocampus

**FIGURE 9:**
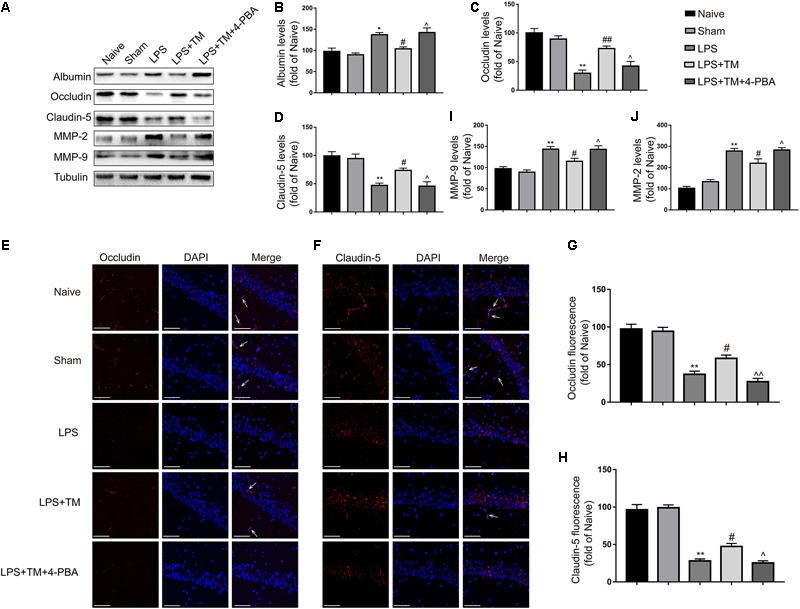
Mild ER stress attenuated LPS-induced BBB hyperpermeability in hippocampus. **(A)** The expression levels of albumin, occludin, claudin-5, MMP-2, and MMP-9 were detected in the hippocampus of rats by Western blotting using specific antibodies. **(B–D)** Expression of albumin, occludin and claudin-5 was quantified and normalized to Tubulin levels. **(E,F)** Images acquired by confocal microscopy show the occludin and claudin-5 levels in the CA1 area of the hippocampus. The arrow in G points to an area in the CA1 area with high occludin immunoreactivity. The arrow in H points to an area in the CA1 area with high claudin-5 immunoreactivity. Scale bar, 100 μm. **(G,H)** Quantitative data of the mean intensities of occludin and claudin-5 fluorescence. **(I,J)** Expression of MMP-2 and MMP-9 was quantified and normalized to Tubulin levels. Each value was expressed relative to the values of the naïve group, which was set to 100 (*n* = 6). The data are representative of three independent experiments. ^∗^*P* < 0.05, ^∗∗^*P* < 0.01 vs. naïve group. ^#^*P* < 0.05, ^##^*P* < 0.01 vs. LPS treatment group. ^∧^*P* < 0.05, ^∧∧^*P* < 0.01 vs. TM treatment group. The data are presented as the mean ± SEM.

As BBB disruption leads to an extravasation of blood-borne proteins, we examined the leakage of albumin to confirm the integrity of the BBB. As shown in **Figure 9A**, LPS significantly increased albumin levels in the hippocampus of rats, compared with the naive group. Although TM administration induced a dramatic decrease in albumin expression, 4-PBA greatly diminished this effect (**Figure 9B**).

#### Mild ER Stress Inhibited the Decreases in Hippocampal Occludin and Claudin-5 Induced by LPS

Decreased tight-junction protein expressions are associated with alterations in BBB permeability. As occludin and claudin-5 are reportedly integral tight-junction membrane proteins (Qi et al., 2016), we examined the levels of occludin and claudin-5 in the hippocampus of rats with western blot analysis. LPS evoked significant decreases in occludin and claudin-5 protein expression compared with the naive group. Treatment with TM effectively negated the increases in hippocampal occludin and claudin-5 induced by LPS, while no elevated occludin and claudin-5 expression was detected after 4-PBA administration (**Figures 9C,D**).

Immunofluorescence staining was also used to analyze occludin and claudin-5 protein expression (**Figures 9E–H**), showing a marked decrease in occludin and claudin-5 protein levels in the LPS group compared with the naive group. Impressively, TM treatment significantly attenuated the occludin and claudin-5 protein expression decrease induced LPS, but 4-PBA cotreatment partially reversed the effect of TM. These results show that mild ER stress prevented BBB disruption by decreasing tight-junction protein levels.

#### Mild ER Stress Reversed the Increases in Hippocampal Matrix Metalloproteinase (MMP)-2 and MMP-9 Induced by LPS

Based on the recent observation that MMP activity is associated with the degradation of tight-junction proteins (Tasaki et al., 2014), we next monitored the expression of MMP-2 and MMP-9 upon mild ER stress induction. As expected, LPS treatment significantly increased MMP-2 and MMP-9 activity, while pretreatment with TM significantly attenuated LPS treatment-induced MMP-2 and MMP-9 activity. The expression levels of MMP-2 and MMP-9 were markedly increased after 4-PBA cotreatment, leading to significant reversal of these TM-evoked effects (**Figures 9I,J**). These results indicate that mild ER stress can inhibit LPS-induced MMP-2 and MMP-9 activation.

#### Mild ER Stress Reduced EB Extravasation in the LPS-Treated Rats

**FIGURE 10:**
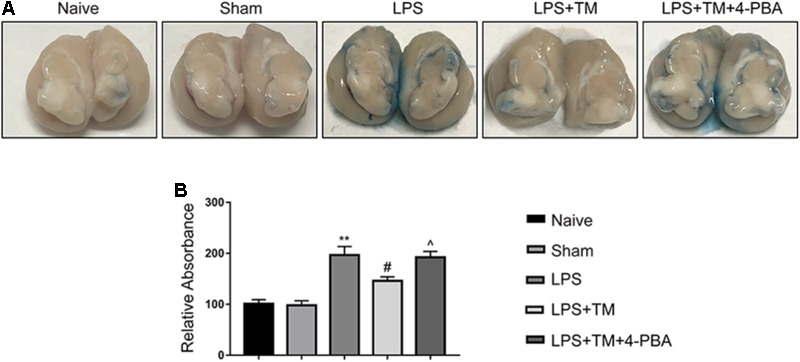
Mild ER stress reduced EB extravasation in the LPS-treated rats. **(A)** Representative photographs of EB extravasation in the extracted brains of various groups. **(B)** The quantitative analysis of EB leakage. Each value was expressed relative to the values of the naïve group, which was set to 100 (*n* = 4). The data are presented as the mean ± SEM. ^∗^*P* < 0.05, ^∗∗^*P* < 0.01 vs. naïve group. ^#^*P* < 0.05, ^##^*P* < 0.01 vs. LPS treatment group. ^∧^*P* < 0.05, ^∧∧^*P* < 0.01 vs. TM treatment group.

EB extravasation is a widely used marker for detecting breaches in the BBB. **Figure 10A** shows the EB extravasation in the extracted brains, indicating BBB disruption. As shown in **Figure 10B**, EB absorbance was increased in the LPS-treated rats compared with the naive group, which was apparently attenuated by TM treatment. Although TM alleviated LPS-induced BBB hyperpermeability, 4-PBA greatly diminished this effect as evidenced by increased EB content in the brain after 4-PBA administration.

## Discussion

Neurodegenerative diseases, exemplified by AD, PD, amyotrophic lateral sclerosis, and multiple sclerosis, pose the most pressing health and economic burden on developed societies with aging populations (Estes and McAllister, 2016). Neuroinflammation is an amplifier of neurodegenerative pathology, and thus, its influence is a subject of increasing interest (Glass et al., 2010).

In recent years, astrocytes have gained attention due to their pivotal role in brain homeostasis both in normal and pathological conditions (Haim and Rowitch, 2017). Long thought of as just supporting cells in the brain, astrocytes have in fact several housekeeping functions. Recently, astrocytes have become the focus of attention because of their metabolic coupling with neurons and the BBB (Cabezas et al., 2014).

The emerging evidence highlights the critical roles of astrocytes in regulating neuroinflammation through a process called “astrogliosis,” (Sofroniew, 2015), which leads to an increase in the number, morphology, and motility of astrocytes and illustrates the role of the neuroinflammatory response in neurodegenerative diseases (Garwood et al., 2017). Astrogliosis is a well-characterized spectrum of cellular, molecular, and functional astrocytic changes in response to CNS damage. The cells in which astrogliosis occurs have the potential to be strongly reactive, thus contributing to the production of numerous proinflammatory molecules, such as cytokines, to exacerbate neuroinflammation (Khakh and Sofroniew, 2015; Bazargani and Attwell, 2016).

In recent years, numerous studies have shown that ER stress in astrocytes is associated with astrogliosis and the development of neuroinflammation (Cheng et al., 2013; Hong et al., 2016). However, these studies, which utilized high concentrations of pharmacological ER stressors to cause robust perturbation of ER function in experimental animals or cultured cells, failed to reproduce physiological and non-lethal levels of ER stress. ER stress activates the signaling events termed UPR. UPR is a heterogeneous and context-dependent cellular response, determined by the intensity or duration of the exposure to stress (Hetz and Mollereau, 2014). Under severe and robust ER stress, activation of the UPR operates as a pro-apoptotic program that reinforces the expression of pro-apoptotic components such as CHOP through ATF4; ER-related neurotoxicity consequently occurs. Under conditions of moderate and mild ER stress, ATF4 and CHOP are highly unstable at the mRNA and protein levels; these proteins are necessarily short-lived without a persistent and robust ER-stress signal transduction pathway (Matus et al., 2014). Therefore, mild perturbations of ER function may operate as an adaptive feedback mechanism of resistance against any possible further injuries.

We evaluated the levels of ATF4 and CHOP expression in astrocytes to further characterize the extent of ER stress in response to varying concentrations of TM. In the present investigation, activation of the PERK-EIF2α/IRE1-XBP1 pathway was detected at both high and low concentrations of TM. However, TM at the highest dose triggered a robust ER-stress response in astrocytes. Only at high concentrations (10 ng/ml) was TM able to trigger a robust ER-stress response in astrocytes, as evident by effective upregulation of ATF4 and CHOP, while lower doses of TM did not increase CHOP and ATF4 protein expression.

GFAP overexpression is a reliable marker for identifying reactive astrocytes under pathological conditions in the hypertrophic response of astrogliosis (Allaman et al., 2011). In this study, we observed that LPS altered the morphology of astrocytes, as demonstrated by terminal swelling filopodium-like processes, and induced the upregulation of GFAP expression. Additionally, LPS significantly induced the inflammatory cytokines, IL-1β, and IL-6, in cultured primary astrocytes. We defined *in vitro* and *in vivo* experimental conditions in which mild ER stress did not induce astrocytic lethality but rather suppressed LPS-induced overactivation and inflammatory responses in astrocytes. We demonstrated that astrocyte protection occurred as an intrinsic consequence of non-toxic, mild activation of ER stress.

It was reported that the heterogeneity between mild ER stress and robust ER stress pertains to differences in the expression of downstream proteins. Although less is known about this non-toxic ER stress in various models, current information points toward a concept that describes this adaptive reaction, which we refer to as “hormesis” (Matus et al., 2014). For instance, TM at a low dosage orchestrated neuroprotection in *Drosophila* and mouse models of PD (Fouillet et al., 2014), AD (Casas-Tinto et al., 2011), brain inflammation (Hosoi et al., 2014), and brain ischemia/reperfusion (Ibuki et al., 2012).

Our previous study revealed that preconditioning with the ER stress-inducer, TM, at a low dosage protected against LPS-induced cognitive decline and neuroinflammation, while 4-PBA partly counteracted this protective effect (Wang et al., 2017). We have proposed that ER-mediated hormesis (or ER hormesis) is responsible for protection after LPS challenge. This hypothesis is also supported by our current results that showed that mild doses of TM relieved LPS-stimulated BBB hyperpermeability.

Albumin is a vascular marker that crosses the BBB very slowly and is therefore often used to measure the loss of BBB integrity (Xaio et al., 2001). Using the albumin quota, we confirmed the role of mild ER stress in BBB function. We found more albumin leakage in the hippocampus following LPS injection, which was inhibited by pretreatment with TM. Furthermore, our study showed that TM treatment alleviated BBB dysfunction and hyperpermeability, evident by the increased levels of tight-junction proteins claudin-5 and occludin along with reduced expression of MMP-2 and MMP-9. It is becoming clear that, on one hand, astrocytes can exert proinflammatory effects by releasing molecules that enhance BBB permeability; on the other hand, however, astrocytes can promote BBB-structure repair by releasing trophic factors (Cabezas et al., 2014). It is difficult to determine the specific mechanism underlying the astrocytic influence on the BBB in our experiment. Further detailed work is required to address this issue in the future.

In summary, our results support a potential protective role of mild ER stress in alleviating LPS-induced astrocytic overactivation and BBB disruption. The central feature of this protective mild ER stress appears to be the triggering and maintenance of ER stress at a moderate level that facilitates survival, without increasing the levels of pro-apoptotic proteins such as CHOP and ATF4. While many crucial questions remain to be answered, our study findings provide not only a better understanding of the role of ER stress in neurodegeneration but also the basis for a prospective cure for neurodegeneration in the future.

## Author Contributions

YC, QZ, JX, QQ, and PN performed the experiments. YW and YQ designed the study. YW wrote the manuscript. All authors read and approved the final manuscript.

## Conflict of Interest Statement

The authors declare that the research was conducted in the absence of any commercial or financial relationships that could be construed as a potential conflict of interest.
