# Supplementary material for: Mild Endoplasmic Reticulum Stress Protects Against Lipopolysaccharide-Induced Astrocytic Activation and Blood-Brain Barrier Hyperpermeability
Source: Front Cell Neurosci. 2018 Jul 27;12:222. doi: 10.3389/fncel.2018.00222 (PMC6077757; doi:10.3389/fncel.2018.00222)
Supplement: Supplementary file 1 [file Data_Sheet_1.PDF]

Supplementary material

Reagents and antibodies

Dulbecco's modified Eagle's medium (DMEM), 0.25% trypsin-EDTA solution and fetal calf serum (FCS) were purchased from Gibco-BRL (Grand Island, NY, USA). Tunicamycin (TM), 4-phenylbutyric acid (4-PBA), and lipopolysaccharide (LPS, from *Escherichia coli*, 0111:B4) were purchased from Sigma-Aldrich (St. Louis, MO, USA). Antibodies targeting total protein kinase RNA-like ER kinase (t-PERK), and phospho(T981)-PERK (p-PERK) were purchased from Santa Cruz Biotechnology (Santa Cruz, CA, USA). Antibodies against phosphor(S724)- inositol-requiring enzyme 1α (IRE1α), total IRE1α, activating transcription factor (ATF)-4, glial cell-derived neurotrophic factor (GDNF), matrix metalloproteinase (MMP)-2, MMP-9, occludin, vascular cell adhesion molecule 1(VCAM-1) and Fluoroshield Mounting Medium with 4',6-diamidino-2-phenylindole (DAPI) were purchased from Abcam (Hong Kong, China).Antibodies against CCAAT/enhancer-binding protein-homologous protein (CHOP), total EIF2α (t-EIF2α), albumin, glial fibrillary acidic protein (GFAP), phosphor (S51)-EIF2α (p-EIF2α), spliced X-box binding protein-1 (XBP1s), XBP1u and goat anti-mouse secondary antibodies and goat anti-rabbit secondary antibodies were obtained from Cell Signaling Technology (Beverly, MA, USA). An antibody against claudin-5 was purchased from Invitrogen (Carlsbad, USA). A rat interleukin-6 (IL-6) enzyme-linked immunosorbent assay (ELISA) kit and rat IL-1β ELISA kit were obtained from eBioscience (San Diego, USA). RIPA buffer, Cell Counting Kit-8 (CCK-8) kit and the BCA kit were obtained from Beyotime (Shanghai, China).

Trace fear conditioning (TFC)

Hippocampal-dependent memory in rodents was evaluated by trace fear conditioning (TFC) as previously described (Almolda et al. 2015, Feng et al. 2013, Terrando et al. 2015). Rats were trained to associate an environment (context) with a conditional stimulus (tone) and an unconditional stimulus (foot shock). The training consisted of placing the rat in the conditioning chamber and allowing exploration of the surroundings for 100 s. Next, the conditional stimulus—an auditory cue (80 dB, 5 kHz)—was presented for 20 s. The unconditional stimulus, a 2-s foot shock (0.8 mA), was administered after termination of the tone. This procedure was repeated with an interval of 100 s, and the rats were removed from the chamber 30 s later. Contextual assessment was performed 24 h after surgery in the same chamber but with no cues (tone or shock). Freezing behavior, recognized as lack of movement, was recorded for 300 s by video and analyzed using software (Xeye Fcs, Beijing MacroAmbition S&T Development Co., Ltd., Beijing, China). A decrease in the percentage of time spent frozen indicated impairment of memory.

Y-maze test

The Y-maze test was used to assess spatial working memory in rodents as previously described (Lu et al. 2015). The Y-maze consisted of three identical arms (30 ×5 × 20 cm). Each arm had a lamp at the distal end. A safe region was associated with the

illumination, whereas the other regions featured electrical foot stimulation (40 ± 5 V). One arm was randomly selected as the "start" arm. The rat was put into the end of the "start" arm (starting area chosen randomly) and allowed to explore the maze freely for 3 min. The test was then started, and the illuminated arm (safe region) served as the new starting area. Furthermore, we randomly changed the orientation of the safe and stimulation regions. The test was considered successful when all four paws of the rats reached the safe region within 10 s. After each foot stimulation, we waited for the rat to reach the illuminated arm (the new starting area) before the next stimulation. If nine responses were correct in ten consecutive foot stimulations (9/10 standard), the rats were classified as having functional working memory. The total number of stimulations to reach the criterion during training was recorded as the learning ability. All rats satisfied the learning criterion in the present study.

Enzyme-linked immunosorbent assay (ELISA)

Cytokine quantification in the hippocampus and microglia was assessed by ELISA using commercial kits for IL-1β and IL-6 according with the manufacturer's instructions. Briefly, 96-well microplates were sensitized with the primary antibody at room temperature (RT) for 30 min, and then the samples were added and incubated at 37°C for 30 min. After the samples were washed, secondary antibody conjugated with peroxidase was added and incubated. The cytokine concentrations were spectrometrically determined using a micro ELISA reader.

References

Almolda, B., de Labra, C., Barrera, I., Gruart, A., Delgado-Garcia, J. M., Villacampa, et al. (2015). Alterations in microglial phenotype and hippocampal neuronal function in transgenic mice with astrocyte-targeted production of interleukin-10. Brain Behav. Immun. 45**,** 80-97.doi: 10.1016/j.bbi.2014.10.015

Feng, X., Degos, V., Koch, L. G., Britton, S. L., Zhu, Y., Vacas, S., et al. (2013). Surgery results in exaggerated and persistent cognitive decline in a rat model of the Metabolic Syndrome. Anesthesiology.118**,** 1098-105.doi: 10.1097/ALN.0b013e318286d0c9

Lu, S., Yu, C., Liu, Y., Dong, H., Zhang, X., Zhang, S., Hu, L., et al (2015). S100A8 contributes to postoperative cognitive dysfunction in mice undergoing tibial fracture surgery by activating the TLR4/MyD88 pathway. Brain Behav. Immun. 44**,** 221-34.doi: 10.1016/j.bbi.2014.10.011

Terrando, N., Yang, T., Ryu, J. K., Newton, P. T., Monaco, C., Feldmann, M., et al (2015). Stimulation of the alpha7 nicotinic acetylcholine receptor protects against neuroinflammation after tibia fracture and endotoxemia in mice. Mol. Med.20**,** 667-75.doi: 10.2119/molmed.2014.00143

Supplementary Figure 1

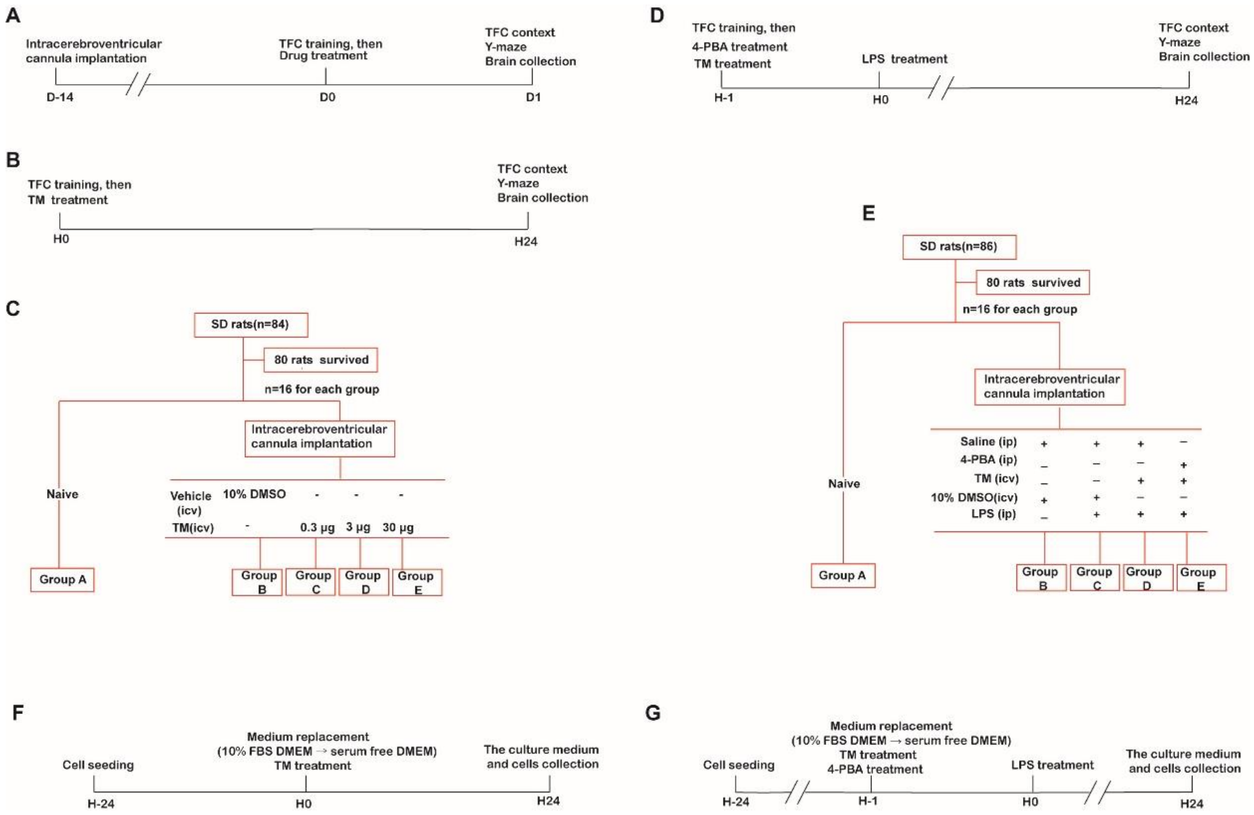

Supplementary Figure 1 Study design. **(A)** Timeline of the in vivo experimental treatments. All rats underwent intracerebroventricular cannula implantation 14 days before use in experiments. One day after contextual fear conditioning training, all animals received drug treatments as indicated. Brains were collected 24 h after drug injection. Contextual assessment and the Y-Maze test were also performed at this time point. **(B and C)** The protocol performed in the in vivo experiment one: Groups C, D and E received the indicated dosage of tunicamycin (TM) intracerebroventricularly immediately after contextual fear conditioning (TFC) training, while group B received an equivalent volume of vehicle. Rats of group A were naïve to all treatments. **(D and E)** The protocol performed in the in vivo experiment two: Rats in groups C, D and E were injected with LPS within 1 h after TFC training. Groups D and E received TM intracerebroventricularly immediately after TFC training, while groups B and C received an equivalent volume of vehicle. 4-PBA was administered by intraperitoneal (ip) injection prior to TM treatment in group E. Rats of group A were naïve to all treatments. All animals underwent behavioral testing 24 h after TM injection. Brains were collected after completion of the behavior tests. **(F)** The protocol performed in the in vitro experiment one: cells were treated with TM at the concentrations indicated for 24 h, then the culture medium and cells were collected. **(G)** The protocol performed in the in vitro experiment two: cells were pretreated with TM for 1 h followed by the addition of LPS. The culture medium and cells were collected after 24 h of incubation.
